# Accelerometer profiles of physical activity and inactivity in normal weight, overweight, and obese U.S. men and women

**DOI:** 10.1186/1479-5868-7-60

**Published:** 2010-08-03

**Authors:** Catrine Tudor-Locke, Meghan M Brashear, William D Johnson, Peter T Katzmarzyk

**Affiliations:** 1 Pennington Biomedical Research Center, Baton Rouge, LA, 70808, USA

## Abstract

**Background:**

The 2005-2006 National Health and Nutrition Examination Survey (NHANES) is used to describe an accelerometer-derived physical activity/inactivity profile in normal weight (BMI < 25 kg/m^2^), overweight (25 ≤ BMI < 30 kg/m^2^), and obese (BMI ≥ 30 kg/m^2^) U.S. adults.

**Methods:**

We computed physical activity volume indicators (activity counts/day, uncensored and censored steps/day), rate indicators (e.g., steps/minute), time indicators (employing NHANES activity counts/minute cut points to infer time in non-wear, sedentary, low, light, moderate, and vigorous intensities), the number of breaks in sedentary time (occasions when activity counts rose from < 100 activity/counts in one minute to ≥ 100 activity counts in the subsequent minute), achievement of public health guidelines, and classification by step-defined physical activity levels. Data were examined for evidence of consistent and significant gradients across BMI-defined categories.

**Results:**

In 2005-2006, U.S adults averaged 6,564 ± SE 107 censored steps/day, and after considering non-wear time, they spent approximately 56.8% of the rest of the waking day in sedentary time, 23.7% in low intensity, 16.7% in light intensity, 2.6% in moderate intensity, and 0.2% in vigorous intensity. Overall, approximately 3.2% of U.S. adults achieved public health guidelines. The normal weight category took 7,190 ± SE 157 steps/day, and spent 25.7 ± 0.9 minutes/day in moderate intensity and 7.3 ± 0.4 minutes/day in vigorous intensity physical activity. The corresponding numbers for the overweight category were 6,879 ± 140 steps/day, 25.3 ± 0.9 minutes/day, and 5.3 ± 0.5 minutes/day and for the obese category 5,784 ± 124 steps/day, 17.3 ± 0.7 minutes/day and 3.2 ± 0.4 minutes/day. Across BMI categories, increasing gradients and significant trends were apparent in males for sedentary time and decreasing gradients and significant trends were evident in time spent in light intensity, moderate intensity, and vigorous intensity. For females, there were only consistent gradients and significant trends apparent for decreasing amounts of time spent in moderate and vigorous intensity.

**Conclusions:**

Simple indicators of physical activity volume (i.e., steps/day) and time in light, moderate or vigorous intensity physical activity differ across BMI categories for both sexes, suggesting that these should continue to be targets for surveillance.

## Background

The 2005-2006 National Health and Nutrition Examination Survey (NHANES) data indicate that more than one-third of U.S. adults are obese (BMI ≥ 30 kg/m^2^), including 33.3% of men and 35.3% of women [1]. A potential contributor to this state of affairs is reduced physical activity. However, self-reported participation in leisure-time physical activity has remained relatively stable over time [2] and more recently (between 2001 and 2005) appears to have increased slightly [3]. Although it is easy to conclude that this apparent paradox points solely to dietary overconsumption as the driving force behind the obesity epidemic [4], it may also be explained by the fact that gains in leisure-time physical activity might not compensate sufficiently for the diminishing alternative opportunities for expending energy. Specifically, there has been a noticeable secular transition in work-related physical activity demands (moving increasingly from physical labor to sedentary occupations) [5], domestic mechanization through labor-saving devices [6], short-distance transportation modes and patterns (moving increasingly to motorized travel from non-motorized travel), and a persistent predilection for passive recreational pursuits (including sustained record levels of television viewing behaviors, despite competition from other electronic media [7]).

Most experts agree that technological advances have reduced lifestyle activity,[8-10] however, there are precious few direct data to support this. Perhaps the best example is a study of Old Order Amish who reject motorized technologies and practice a traditional lifestyle of 'living off the land'[11]. Men from this group take approximately 18,000 steps/day and women take 14,000 pedometer-determined steps/day. This is in stark contrast to data collected in two U.S. pedometer-based samples: Colorado (≅6,800 steps/day)[12] and South Carolina (≅ 5,900 steps/day)[13]. Since the discrepancy between these Amish (reflective of an earlier pre-technology time) and more contemporary values ranges between 7,000 to 12,000 steps/day, it is likely that the erosion of daily steps began earlier in the past century, but it is only as these transitions have proliferated and compounded, reaching a "tipping point" in the 1980s in the USA, that we have been able to trace obvious indicators of the increasing obesity epidemic. Developing nations are now experiencing a similar process [14].

Considering these diverse behavioral suspects, it is necessary to capture a full range of physical activity/inactivity estimates concurrently in normal weight, overweight, and obese individuals if we are to better establish a comprehensive physical activity profile [15] associated with obesity. The concept of physical activity profiling (e.g., examining a complete panel of physical activity/inactivity indicators simultaneously) attempts to move beyond single point estimates of time spent only in moderate-to-vigorous physical activity (MVPA), for example [15,16]. Physical activity profiling is an essential first step to teasing out which aspects of a full physical activity spectrum may most likely be contributing to the growing obesity problem and can also point the way to a minimal data set necessary for surveillance purposes.

Objective monitoring by accelerometry produces a wealth of information that can be distilled and examined to better understand the complex nature of a full range of human physical activity/inactivity behaviors. For example, NHANES now uses accelerometers to objectively capture free-living physical activity behaviors as part of its surveillance system. The accelerometer used (Actigraph AM-7164) produces raw outputs of minute-by-minute steps (depicting a movement event; released for the first time in the 2005-2006 NHANES) and activity counts (denoting acceleration of movement in addition to its occurrence; available since the 2003-2004 NHANES). These raw activity counts can be transformed to produce a number of indicators of physical activity/inactivity. For example, since the ActiGraph data are time-stamped, it is possible to derive duration spent in different intensities of activity (including sedentary time) using activity counts/minute cut points (e.g., those established for the NHANES data) [17]. The 2003-2004 NHANES accelerometer data have been used to identify prevalence of achieving minimal public health guidelines (based on intensity bouts and their duration) [17]. The same data were used to analyze time in sedentary behaviors, defined as time spent < 100 activity counts/minute [18]. We have recently used the 2005-2006 data to provide population and sex-specific epidemiology of accelerometer-determined steps/day with and without censoring steps detected at very low intensity [19]. Accelerometer data can also be manipulated to express physical activity as a summed volume of activity counts engaged in over the course of the day or as a rate (either activity counts/minute or steps/minute) considering time worn [20]. Recently, Healy et al. [21] have demonstrated that breaks in sedentary time transitions (e.g., moving from sitting to standing) can be counted using ActiGraph data and may be associated with metabolic risk.

The opportunity at hand is to describe a comprehensive accelerometer-derived physical activity/inactivity profile in body mass index (BMI) defined normal weight, overweight, and obese men and women using the 2005-2006 NHANES data. Although BMI has recognized limitations as an indicator of body fatness/obesity (e.g., it overestimates body fat in muscular individuals and can underestimate body fat in those who have lost muscle mass), it is a favoured measure of excess weight in epidemiological studies focused on relative risk of disease [22].

## Methods

### NHANES 2005-2006

NHANES began in the early 1960s as a periodic health survey and became continuously implemented since 1999. Approximately 5,000 people engage in interviews and physical examinations each year and the data are released in two-year intervals. The physical activity monitor component was added to NHANES in 2003 to objectively assess participants ≥ 6 years of age. The 2005-2006 survey marks the first release of accelerometer-determined step data in addition to the more commonly evaluated output of time-stamped activity counts/minute, making it the most inclusive ActiGraph data set available on a large surveillance study (nationally representative of the U.S.).

NHANES contains data from individuals selected under a complex, multistage probability design to be representative of the civilian, non-institutionalized U.S. population. Households are identified, interviews are conducted in the home, and participants are subsequently invited to a mobile examination center to receive a health examination. Participants with no walking impairments (or other limitations that prevent wearing an accelerometer) are invited to wear the accelerometer and instructed in its standard use. The device, programmed to record information each minute, is worn on the right hip using an elasticized fabric belt. Standardized instructions include wearing the accelerometer during waking hours for 7 days and only removing it for water activities such as swimming, showering, and bathing. Accelerometers are returned to the NHANES data center by pre-paid mail and participants are compensated $40.

The 2005-2006 data file of minute-by-minute activity count and step data was made publically available at http://www.cdc.gov/nchs/nhanes.htm in June, 2008. The data set was prepared for release by NHANES staff who reviewed it for unreasonable values, for example, occurrences of zero steps and > 250 activity counts/minute (Captain Richard P. Troiano, personal communication) and whether or not instruments remained calibrated upon their return. Reliable data are clearly indicated for ready use. The National Center for Health Statistics (NCHS) Ethics Review Board approved the original survey protocols, and informed consent was obtained for all NHANES participants. The Pennington Biomedical Research Center's Institutional Review Board approved of this analysis.

### Data Treatment

Accelerometer time worn (hours and minutes) was computed using a specifically designed SAS macro provided by the National Cancer Institute (NCI) at http://riskfactor.cancer.gov/tools/nhanes_pam/. This macro infers non-wear from at least 60 contiguous minutes of zero activity count data points. This decision rule results in a more comprehensive data set when compared to 20 minutes, for example [23].

This analysis was focused on adult participants ≥ 20 years of age. Of the 4,372 eligible men and women in the sample, we excluded 171 with NHANES-designated unreliable data and 185 with accelerometers determined not to be in calibration upon return. Since this analysis focused on BMI-defined weight categories, we excluded 269 women who self-reported pregnancy and a single individual with a BMI > 100 kg/m^2^. We also excluded 224 individuals who did not have at least one valid day of wear (defined as ≥ 10 hours of wear [17-19]), in keeping with previous analyses [18,19]. A more thorough discussion of the appropriateness of using a single day of wear is available elsewhere [19]. Ultimately, this analysis is based on 3,522 individuals or 81% of the originally eligible sample. We did not present a summary of our results on accelerometer data for men and women with a ≤ 18.5 kg/m^2 ^(technically considered underweight) because such data were available only for 69 adults, a sample size we feel is too small for informative interpretation.

Table 1 summarizes the panel of indicator variables comprising a comprehensive accelerometer-derived physical activity/inactivity profile of U.S. men and women. All indicator variables have been previously defined and used in the literature; we did not create anything new for this specific analysis. Physical activity volume indicators included daily totals of activity count and step data averaged over the number of days worn to produce activity counts/day and steps/day, respectively. Following previously published methods [19], steps/day were further transformed after censoring out those steps taken at an intensity < 500 activity counts/minute (equivalent to low intensity and sedentary behaviors, defined further below). Since the ActiGraph is known to be more sensitive to low force movements than accepted research quality pedometers, leading to relatively higher step estimates [20,24,25], this censoring manipulation was necessary to bring the accelerometer-determined steps/day more in line with current pedometer-based scales [26] and comparable surveillance studies [12,13,27]. The process only affects step data. Time in intensity derived from the activity count data is unaffected. In this analysis we present both uncensored (i.e., raw) and censored steps/day.

**Table 1:** Accelerometer physical activity/inactivity indicator variables

Accelerometer variable	Defined
Volume indicators*	
Activity counts/day	Total activity counts accumulated over 1,440 minutes
Uncensored steps/day	Total raw steps accumulated over 1,440 minutes
Censored steps/day	Total steps accumulated over 1,440 minutes after censoring out those steps taken at an intensity < 500 activity counts/minute
Rate indicators*	
Activity counts/minute	Total activity counts accumulated over 1,440 minutes, divided by time worn
Uncensored steps/minute	Total raw steps accumulated over 1,440 minutes (24 hours or one day), divided by time worn
Censored steps/minute	Total steps accumulated over 1,440 minutes after censoring out those steps taken at an intensity < 500 activity counts/minute, divided by time worn
Time indicators (minutes)*	
Non-wear time	1,440 minutes minus wear time
Time in sedentary intensity	Total time < 100 activity counts/minute
Time in low intensity	Total time 100-499 activity counts/minute
Time in light intensity	Total time 500-2,019 activity counts/minute
Time in moderate intensity	Total time 2,020-5,999 activity counts/minute
Time in vigorous intensity	Total time > 5,999 activity counts/minute
**Achievement of public health guideline indicator**	
Y/N achievement of public health guidelines	Percent achieving ≥ 30 minutes of at least moderate intensity in modified 10-minute bouts** on at least 5 of 7 days
Break in sedentary time indicator*	
Transitions/day	Total occurrences of when activity counts rose from < 100 activity/counts in one minute to ≥ 100 activity counts in the subsequent minute
**Step-defined activity levels (%)**	
Basal activity	< 2,500 censored steps/day
Limited activity	2,500 to 4,999 steps/day
Low active	5,000-7,499 steps/day
Somewhat active	7,500-9,999 steps/day
Active	10,000-12,499 steps/day
Highly active	≥ 12,500 steps/day

*averaged over number of days worn

**10 or more consecutive minutes above the moderate intensity activity count threshold, with allowance for interruptions of 1 or 2 minutes below threshold. A bout was terminated by 3 minutes below threshold.

Physical activity rate indicators included activity counts/minute, uncensored steps/minute, and censored steps/minute, each computed considering time worn. Each of the daily 1,440 minutes recorded for each individual were categorized by intensity according to specific activity counts/minute values that have been previously used to analyze NHANES data [17] to produce a series of time indicators. In particular, 2,020 activity counts/minute is considered the threshold value indicative of moderate intensity activity and 5,999 activity counts/minute stands for minimally vigorous intensity activities. Time spent at < 100 activity counts/minute (after removing non-wear time was identified as engaging in sedentary behaviors [18]. Non-wear time (which logically includes sleep time) represents the difference between 1,440 minutes and wear time (that is, wear time is calculated by subtracting macro-determined non-wear time from 1,440). Time spent at < 2,020 activity counts/minute) was further segmented into low (100-499 activity counts/minute) and light (500-2,019 activity counts/minute) intensity activities consistent with previous analyses that have looked at finer gradations of intensity categories simultaneously [20]. In that previous study, activity counts/minute < 500 were originally labeled "inactive" intensity; for clarity and consistency we have chosen to re-label it here "low" intensity. Daily minutes in each intensity category were summed. Total daily minutes within intensity categories were summed and subsequently divided by the number of days worn to compute daily average time spent in non-wear, sedentary behaviors, and low, light, moderate, and vigorous intensity activities.

We accessed another NCI-supplied SAS macro (located at http://riskfactor.cancer.gov/tools/nhanes_pam/) to determine achievement of public health guidelines to accumulate 30 minutes of at least moderate intensity in modified 10-minute bouts on at least 5 of 7 days using a Bayesian approach to interpret information from all individuals with one or more valid days [17]. A modified 10-minute bout was defined as 10 or more consecutive minutes above the moderate intensity activity count threshold, with allowance for interruptions of 1 or 2 minutes below threshold. In keeping with previous analyses, a bout was terminated by 3 minutes below threshold [17].

The number of transitions or breaks in sedentary time were counted as occasions when activity counts rose from < 100 activity/counts in one minute to ≥ 100 activity counts in the subsequent minute [21]. These were totalled on a daily basis and averaged over the number of days worn. Finally, we computed the percent of the sample classified using an established step-defined physical activity scale [26,28]: 1) < 5,000 steps/day (sedentary); 2) 5,000-7,499 steps/day (low active); 3) 7,500-9,999 steps/day (somewhat active); 4) 10,000-12,499 steps/day (active); and 5) ≥ 12,500 steps/day (highly active). We have previously also segmented the pedometer-defined sedentary level into < 2,500 steps/day (basally active) and 2,500 to 4,999 steps/day (limited activity) [19] and the complete set of step-defined physical activity levels are used herein. These categories reflect the use of research quality pedometers, highlighting the need to align the NHANES accelerometer-determined steps data accordingly. Therefore, we used the censored steps/day data for these classifications.

Descriptive statistics (means or frequencies and 95% CIs where appropriate) were calculated for each of the physical activity/inactivity variables and presented by normal weight (BMI < 25 kg/m^2^), overweight (25 ≤ BMI < 30 kg/m^2^), and obese (BMI ≥ 30 kg/m^2^) categories for the total sample and by sex. Data were examined for evidence of consistent gradients across BMI categories (consistent increasing or decreasing pattern vs. a randomly fluctuating pattern) and statistical tests were conducted to evaluate significant trends, also across BMI categories.

## Results

Table 2 summarizes the accelerometer-derived volume, rate, and break in sedentary time indicators for 2005-2006 NHANES U.S. adults. All volume and rate indicators show a clear and significant decreasing gradient across BMI categories, and they were also consistently higher for males compared to those for females. The difference in breaks in sedentary time across BMI categories appears to be minimal (a difference of only 1-2 breaks per day) but does show a similar gradient, however this was only statistically significant in the males. Across BMI categories, however, females consistently took relatively more breaks in sedentary time compared to males.

**Table 2:** Indicators of volume, rate, and breaks in sedentary time: 2005-2006 NHANES U.S. Adults

			Obesity Category		
			
			Normal Weight N = 1016, Males = 461, Females = 555	Overweight N = 1195, Males = 725, Females = 470	Obese N = 1242, Males = 562, Females = 680
			
Accelerometer variable					
Volume indicators**			**Mean (95% CL)**	**Mean (95% CL)**	**Mean (95% CL)**

	**Activity counts/day**	**All**	245743	243106	201958
			(236709, 254776)	(230874, 255340)	(192613, 211303)‡
		**Male**	282211	265224	224755
			(270112, 294311)	(251468, 278979)	(212759, 236751)‡
		**Female**	222335	211356	183030
			(210842, 233827)	(198266, 224446)	(168426, 197635)†
	**Uncensored steps/day**	**All**	10379	10015	8893
			(10032, 10727)	(9661, 10369)	(8606, 9180)‡
		**Male**	11402	10741	9889
			(10928, 11876)	(10330, 11153)	(9580, 10198)‡
		**Female**	9723	8972	8066
			(9319, 10127)	(8543, 9402)	(7684, 8448)‡
	**Censored steps/day**	**All**	7190	6879	5784
			(6856, 7524)	(6581, 7177)	(5520, 6047)‡
		**Male**	8285	7643	6644
			(7829, 8742)	(7301, 7984)	(6383, 6905)‡
		**Female**	6486	5782	5069
			(6091, 6882)	(5417, 6148)	(4703, 5435)‡
Rate indicators*					

	**Activity counts/minute**	**All**	343.9	332.3	287.5
			(332.4, 355.4)	(323.3, 341.2)	(277.8, 297.2)‡
		**Male**	395.3	363.5	314.9
			(375.9, 414.8)	(351.2, 375.8)	(302.4, 327.4)‡
		**Female**	310.9	287.4	264.8
			(297.1, 324.6)	(274.9, 299.9)	(251.2, 278.3)‡
	**Uncensored steps/minute**	**All**	12.3	11.8	10.6
			(11.9, 12.6)	(11.4, 12.1)	(10.3, 10.9)‡
		**Male**	13.3	12.5	11.7
			(12.8, 13.8)	(12.1, 12.9)	(11.3, 12.0)‡
		**Female**	11.6	10.7	9.8
			(11.1, 12.1)	(10.3, 11.2)	(9.4, 10.2)‡
	**Censored steps/minute**	**All**	8.5	8.1	6.9
			(8.1, 8.9)	(7.8, 8.3)	(6.6, 7.2)‡
		**Male**	9.7	8.9	7.8
			(9.2, 10.2)	(8.5, 9.2)	(7.5, 8.2)‡
		**Female**	7.7	6.9	6.1
			(7.2, 8.2)	(6.5, 7.3)	(5.7, 6.6)‡
Break in sedentary time indicator*					

	**Transitions/day**	**All**	92.7	91.5	90.4
			(90.9, 94.6)	(90.2, 92.8)	(89.3, 91.5)†
		**Male**	90.9	90.2	88.4
			(88.5, 93.4)	(88.2, 92.1)	(87.1, 89.8)†
		**Female**	93.9	93.5	92.1
			(92.0, 95.8)	(91.4, 95.5)	(90.4, 93.8)

*averaged over number of days worn

**10 or more consecutive minutes above the moderate intensity activity count threshold, with allowance for interruptions of 1 or 2 minutes below threshold. A bout was terminated by 3 minutes below threshold.

‡p < 0.0001, †p < 0.03 for linear trend

**Figure 1:**
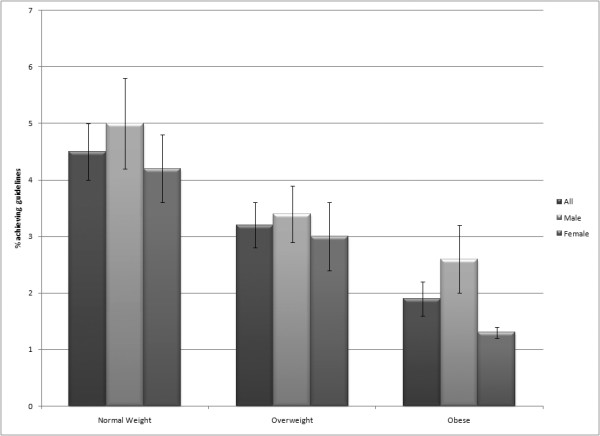
Percent achieving public health guidelines by BMI category (normal weight < 25 kg/m^2^, overweight 25 kg/m^2 ^≤ BMI < 30 kg/m^2^, and obese BMI ≥ 30 kg/m^2^) and sex

Figure 1 displays the percent achieving public health guidelines by BMI category and sex. Overall, achievement of public health guidelines is relatively uncommon (i.e., < 5%). Still, a decreasing gradient is apparent across BMI categories and also between sexes (males > females).

**Figure 2:**
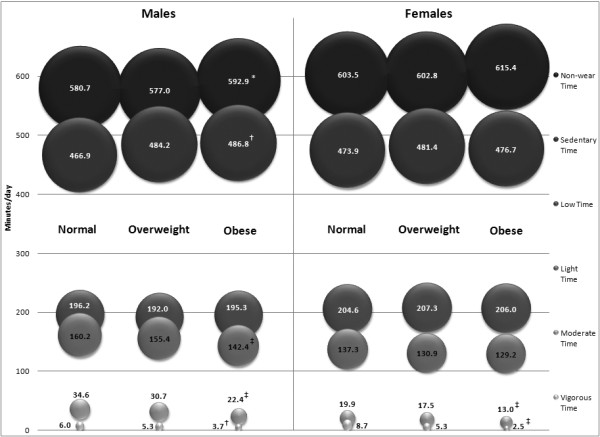
Sex-specific percent of the 1,440 minute day represented by all time indicators across BMI categories. * p < 0.05, † < 0.01, ‡ < 0.001

Figure 2 is a bubble graph that displays daily time spent in each of the time indicators across sex-specific BMI-defined normal weight/overweight/obesity categories. The size and height of the bubble represents the number of minutes/day. Across BMI categories and between sexes the greatest minutes/day were spent, in descending rank order, in non-wear time, sedentary time, low intensity, light intensity, moderate intensity, and vigorous intensity. Although there was no apparent gradient in non-wear time across BMI categories in males, the trend was significant and most obvious for obese males. Time spent in low intensity activities showed no gradient across BMI categories in males, and there was no significant trend. Increasing gradients and significant trends were apparent in males for sedentary time and decreasing gradients and significant trends were evident in time spent in light intensity, moderate intensity, and vigorous intensity. For females, there were only consistent gradients and significant trends apparent for decreasing amounts of time spent in moderate and vigorous intensity. No other time indicators were significant in females across BMI categories. Within BMI categories, males (compared with females) consistently spent less time in non-wear time and in low intensity time and more time in light intensity and moderate intensity across BMI categories. No other differences were consistent.

**Figure 3:**
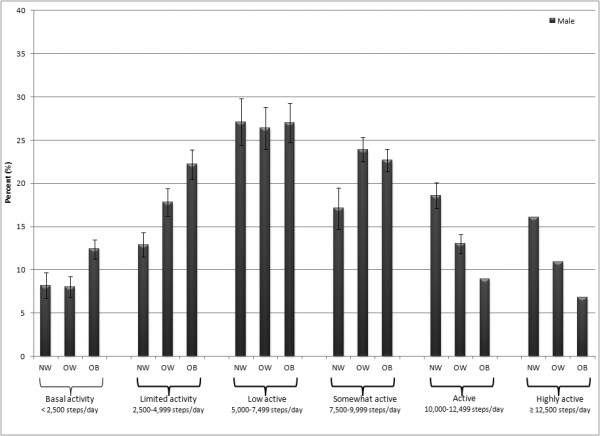
Percent of males classified by step-defined activity levels considering BMI category (normal weight = NW, overweight = OW, obese = OB). Bars depict SE.

Figures 3 (males) and 4 (females) present percents classified by step-defined activity levels. Considering both sexes together, there were relatively more overweight and obese individuals classified as taking basal activity and limited activity. This pattern was reversed in those classified as low active, active, and high active. An exception to these patterns was evident in those classified as somewhat active; relatively more overweight individuals were classified as such compared to either normal weight or obese individuals. By sex, there were relatively more males classified in the somewhat active, active, and highly active categories whereas relatively more females were classified in the basal activity, limited activity, and low active categories.

**Figure 4:**
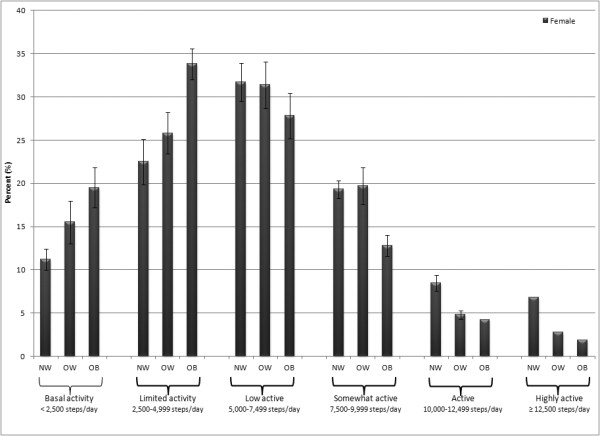
Percent of females classified by step-defined activity levels considering BMI category (normal weight = NW, overweight = OW, obese = OB). Bars depict SE.

## Discussion

This analysis presents the most comprehensive set of accelerometer-derived indicators of physical activity/inactivity using the largest nationally representative data set yet available. As a nation (excepting pregnant women), U.S adults average 6,564 ± SE 107 censored steps/day, and after considering non-wear time, they spend approximately 56.8% of the rest of the waking day in sedentary time, 23.7% in low intensity, 16.7% in light intensity, 2.6% in moderate intensity, and 0.2% in vigorous intensity. Overall, approximately 3.2% of U.S. adults achieve public health guidelines.

Examined across BMI categories for both sexes, a consistent decreasing and statistically significant gradient is apparent for all physical activity volume indicators, as is expected. With regards to time indicators (i.e., measured time spent in various intensity categories), there was a clear moderating effect of sex. Significant gradients were observed for non-wear time, sedentary time, light time, moderate time, and vigorous time (but not low intensity time) in males. For females, only gradients in moderate and vigorous intensity time were significant. Further, despite the fact that achievement of public health guidelines, is, overall, a rare phenomenon (at least as measured by the ActiGraph accelerometer), a decreasing gradient was still evident across BMI categories. Strath et al. [16] have also reported a relationship between time in MVPA and markers of obesity in U.S. adults using the 2003-2004 NHANES data. As this is a cross-sectional analysis, we are not able to conclude whether these decreased physical activity indicators lead to overweight and obesity or whether weight gain reduces these physical activity indicators. Other evidence suggests that both mechanisms are at work on an individual level [29,30]. Body weight status is a complex function of other contributors, including dietary intake, although this is not a focus of this analysis.

The observed gradient in breaks in sedentary time was minimal (a difference of 1-2 breaks/day across BMI categories) and only statistically significant in males. Therefore, although these are only cross-sectional data, these minimal differences would suggest that this specific aspect of sedentary behavior may not be an important contributor to the obesity epidemic [31] (but this does not negate a possible contribution to other important health-related outcomes). Previous reports linking sedentary behavior to obesity have used questionnaire methods to recall sitting time [32,33], but also the same brand and model of accelerometer as used by NHANES [21,34]. We also used the same definitions of sedentary time [34] and breaks in sedentary time [21] as these previous accelerometer-based reports so the very minimal differences we observed cannot be explained by differences in either instrumentation or cut point choices.

We observed a similar decreasing gradient across BMI categories in rate indicators, however, these are ratios and as such need to be interpreted cautiously since they are affected simultaneously by both the numerator and the denominator (in this case, time monitored by accelerometer). Although we observed no consistent differences in non-wear time across BMI categories, variation in either the physical activity volume indicator or time that the accelerometer was worn, or both, can distort conclusions. That being said, a post-hoc analysis of covariance was performed to adjust mean time spent in each intensity for wear time. The results of this analysis were very similar to the unadjusted results. For example, when examining time spent in the sedentary intensity for males, the trend across BMI categories stayed significant with a p-value of 0.0001 compared to the unadjusted p-value of 0.007. Furthermore, the analysis for time spent in the low intensity produced a p-value of 0.64 when adjusted for wear time and a p-value of 0.78 when not adjusted. All categories were examined and were found to produce the same conclusions as the unadjusted model.

Activity counts corresponding to a MET-defined moderate intensity physical activity appear to be much lower in older and overweight/obese populations. Specifically, Lopes et al. [35] conducted an ActiGraph calibration study and determined that 1,240 activity counts/minute represented the threshold for moderate intensity activity in such a population, a value that is less than what has been conventionally used to describe the same intensity behavior in this and other NHANES analyses (i.e., 2020 activity counts/minute) [17]. Physical activity is a behavior and it is quantified herein objectively as steps taken or time above a specific activity count threshold; this threshold captures movement as acceleration. Although conclusions about the metabolic cost of this behavior appear to be affected by factors known to influence energy expenditure (e.g., body mass), we remain nonetheless confident that differences (or lack of differences) between BMI-defined weight categories in objectively-monitored steps taken or their acceleration are real. To emphasize, energy expenditure is higher in obese individuals due to their higher body mass [36]. In terms of physical activity, however, we found that obese individuals (regardless of sex) take fewer steps/day and spend less time in moderate and vigorous intensity activity.

It was not surprising that overweight and obese individuals tended to take fewer steps/day and that normal weight people tended to take more steps/day. As is evident from Figures 3 and 4, within each step-defined activity group, the percent of normal weight, overweight, and obese must add up to 100%; along the physical activity continuum the normal/overweigh/obese gradient "switches" from an upwardly sloping gradient to a downwardly sloping gradient. However, we did not expect to find that more overweight individuals were classified as somewhat active (i.e., taking 7,500-10,000 steps/day) compared to both normal weight and obese individuals. It is plausible that overweight individuals were more likely to modify their physical activity to affect their weight, and then this collective behavior was picked up as a distortion to the expected gradient. A previous analysis of 1-year tracking of pedometer-determined physical activity showed that the percent of obese individuals who increased their physical activity over the previous year was higher than those who decreased their behavior; further, as a group the obese were less stable (that is, more change occurred) in their behavior compared to a normal weight group [37]. The relative instability of physical activity behavior by BMI category requires more research for confirmation.

In keeping with surveillance of reported leisure time physical activity [3] and inactivity [38] that show sex-specific differences, NHANES males were consistently more physically active (i.e., physical activity volume and rate indicators were higher and they spent more time in light and moderate intensity activity) and less physically inactive (i.e., they spent less time in non-wear time and low intensity time) than females across BMI categories. A consistent pattern in sedentary time was not evident between sexes across BMI categories. However, across these same categories, females took relatively more breaks in sedentary time compared to males. There were also relatively more males classified in the somewhat active, active, and highly active step-defined categories whereas relatively more females were classified in the basal activity, limited activity and low active categories. No other patterns (i.e., consistent results between sexes across BMI categories) were apparent.

Acknowledging the limitations of cross-sectional analysis, by scrutinizing a full panel of concurrent estimates of physical activity/inactivity across BMI-defined weight categories, we can begin to identify specific activity parameters that maximally differentiate between normal/overweight/obese samples and therefore best inform on-going surveillance efforts and physical activity interventions. An important caveat to keep in mind, however, is that this was a population analysis and the results do not necessarily apply to all individuals. Causality can only be substantiated, however, in longitudinal and intervention study designs.

## Conclusions

Simple indicators of physical activity volume (i.e., steps/day) and time in moderate or vigorous intensity physical activity differ across BMI categories (regardless of sex), suggesting that these should continue to be targets for surveillance. In addition, longitudinal and intervention designs are needed to confirm a causal role in the obesity epidemic. Despite the current interest in breaks in sedentary time, we found little evidence to support a noteworthy difference in this accelerometer-determined behavior across BMI categories. This does not necessarily negate its impact on other outcomes not examined within the scope of this analysis.

## Competing interests

P.T. Katzmarzyk is supported, in part, by the Louisiana Public Facilities Authority Endowed Chair in Nutrition.

## Authors' contributions

CT-L, WDJ, and PTK worked together to conceive and design the analysis. All authors contributed to interpretation of results, critically revised, edited and read and approved the final version of the manuscript. WDJ led the analysis and data presentation. MMB conducted analyses and data presentation, and provided additional critical input in regards to interpretation and writing of results. CT-L led the writing.
